# Supplementary material for: Studies on the Prediction and Extraction of Methanol and Dimethyl Carbonate by Hydroxyl Ammonium Ionic Liquids
Source: Molecules. 2023 Mar 2;28(5):2312. doi: 10.3390/molecules28052312 (PMC10005580; doi:10.3390/molecules28052312)
Supplement: Supplementary file 1 [file molecules-28-02312-s001.zip › molecules-2189838-supplementary.pdf]

SUPPORTING FILES

Tables

Table S1 S of ILs predicted by COSMO-RS

Table S2 SP of ILs predicted by COSMO-RS

Table S3 SL of ILs predicted by COSMO-RS.

Table S1

anion / cation	PF6	Tf2n	OTF	DMP	2-CP	3-CP	HSO4	NO3	H2PO4	HCO3	Frc	Ac	Prp	BEN	Leu	4FPhO	4-CP	3FPhO	2FPhO	SCN	BF4	BCN4
P4444	0.075	0.115	4.052	12.331	19.755	21.827	1.546	2.083	11.965	15.889	36.502	115.107	120.720	51.358	62.394	48.773	33.485	28.351	27.075	1.243	0.289	0.141
P4442	0.081	0.120	4.165	12.647	19.203	21.112	1.725	2.247	12.767	16.671	37.229	113.993	118.746	50.319	62.121	46.840	32.183	27.463	26.381	1.317	0.323	0.147
N4444	0.078	0.119	4.090	12.514	18.651	20.565	1.640	2.174	12.329	16.303	36.641	111.277	115.436	48.762	60.619	45.368	31.274	26.646	25.606	1.269	0.308	0.145
N2222	0.128	0.150	4.634	13.388	18.167	19.673	2.282	2.542	12.638	14.900	30.229	89.605	101.315	45.222	58.067	41.828	29.173	25.503	24.767	1.594	0.498	0.186
P2221	0.126	0.149	4.626	13.430	17.811	19.277	2.325	2.612	13.029	15.485	31.332	91.123	101.213	44.768	57.851	40.997	28.559	25.006	24.348	1.599	0.500	0.185
MEA	0.174	1.124	9.088	14.944	2.610	2.185	121.919	122.788	145.835	118.308	112.291	64.079	29.650	12.730	23.137	3.667	2.450	3.130	4.216	10.616	3.258	0.782
EMIM	0.240	0.223	4.978	13.412	10.961	11.608	19.955	3.682	13.489	15.083	25.476	56.733	60.288	28.602	40.232	22.077	15.877	14.796	15.197	2.266	1.014	0.295
DEA	1.118	1.650	9.102	10.617	3.216	2.904	26.575	41.176	48.613	44.142	38.692	30.844	19.181	10.150	15.330	4.154	3.107	3.815	4.590	16.349	21.278	1.504
MMIM	0.323	0.239	5.287	13.104	11.349	11.918	3.823	3.279	10.611	10.980	18.176	44.332	53.179	27.629	37.669	22.220	16.129	15.184	15.536	2.356	1.207	0.333
N11116	0.084	0.123	3.104	10.379	10.285	11.108	3.734	2.013	9.219	12.363	25.118	62.915	62.589	27.490	37.247	22.451	15.995	14.088	13.979	1.102	0.329	0.148
P4442OH	0.479	0.490	3.689	11.745	10.305	11.497	1.573	2.558	12.079	15.192	28.152	56.622	53.344	21.803	33.958	23.899	16.594	14.456	14.731	1.349	0.852	0.445
HMIM	0.572	0.443	6.581	13.590	6.623	6.644	2.444	8.947	19.065	18.625	24.773	40.813	38.806	19.858	29.129	12.128	8.446	8.918	9.869	5.616	4.598	0.525
N1111OH	1.893	0.770	6.083	11.279	8.584	8.896	11.889	3.521	7.903	6.985	9.870	21.719	28.512	16.231	23.699	15.949	11.501	11.469	11.962	3.103	3.747	0.842
P4442COOH	1.957	1.478	3.141	10.166	5.342	5.915	5.184	2.750	10.464	12.949	20.967	33.867	30.085	12.260	20.722	11.090	7.993	7.257	7.659	1.373	2.095	1.051
N1111COOH	5.415	2.179	5.893	10.691	5.013	5.047	3.198	7.947	13.625	12.607	15.500	22.698	21.791	11.846	17.896	8.936	6.263	6.704	7.446	5.532	13.433	1.819
TEA	3.182	2.067	7.900	8.558	3.218	3.110	12.593	18.426	21.245	18.918	18.402	18.817	14.662	8.578	11.973	4.225	3.311	3.896	4.381	11.988	20.474	2.097

Table S2

anion / cation	PF6	Tf2n	BCN4	BF4	HSO4	SCN	NO3	OTF	H2PO4	HCO3	DMP	2-CP	3-CP	2FPhO	3FPhO	Frc	4-CP	BEN	4FPhO	Leu	Ac	Prp
N11116	0.291	0.369	0.547	0.747	2.182	2.452	3.202	5.574	7.997	12.606	13.535	20.913	22.425	26.224	27.205	27.981	31.089	41.027	41.298	45.125	66.564	69.069
N2222	0.198	0.320	0.457	0.686	2.427	2.815	4.564	6.796	12.532	24.630	19.969	30.226	32.703	39.969	41.254	65.430	47.947	72.930	67.550	81.564	163.493	146.521
N4444	0.203	0.274	0.443	0.656	2.359	2.948	3.975	7.307	10.963	19.632	20.777	35.494	38.252	46.635	48.558	52.730	56.440	82.033	79.505	89.425	150.847	156.261
P2221	0.204	0.325	0.468	0.682	2.365	2.774	4.379	6.717	11.887	23.066	19.628	29.635	32.080	38.980	40.286	60.750	46.846	70.211	65.610	78.841	152.377	139.250
P4442	0.206	0.282	0.451	0.672	2.397	3.005	4.125	7.634	11.389	20.717	21.698	37.814	40.860	49.735	51.952	56.477	60.603	88.038	85.606	95.495	163.899	169.304
P4442OH	1.791	1.400	1.419	1.496	2.492	2.259	3.251	4.877	7.552	12.638	14.753	15.497	17.009	20.840	20.661	28.172	23.870	27.949	32.988	37.734	55.496	53.648
P4444	0.189	0.257	0.419	0.632	2.364	2.995	4.032	7.544	11.503	20.787	21.817	38.758	41.835	51.349	53.595	57.416	62.547	91.961	89.141	99.321	171.163	178.355
EMIM	0.272	0.506	0.566	0.621	1.739	1.673	2.965	4.079	7.418	13.628	12.615	12.006	13.115	15.063	15.280	30.113	17.582	25.622	22.683	33.763	57.843	50.703
P4442COOH	11.492	5.451	3.665	3.203	2.581	1.923	2.622	3.467	5.186	7.762	9.733	7.600	8.348	9.833	9.636	14.348	10.882	12.477	14.078	17.491	23.190	22.248
MMIM	0.203	0.485	0.494	0.549	1.798	1.593	3.309	3.546	8.848	17.251	12.148	10.622	11.627	13.410	13.504	37.574	15.645	23.809	20.252	32.335	64.030	49.939
N1111COOH	5.860	5.978	3.197	0.804	0.973	0.623	1.195	1.585	3.048	5.123	6.206	3.278	3.780	3.657	3.701	8.511	4.546	5.465	4.932	8.659	11.338	10.209
HMIM	0.580	1.092	0.982	0.333	0.752	0.593	1.215	1.954	3.290	6.003	7.650	4.341	4.967	4.869	5.000	11.439	6.058	8.237	6.702	13.343	17.831	16.357
N1111OH	0.483	1.307	1.046	0.629	1.686	1.207	2.838	2.009	7.446	14.476	8.450	5.365	5.992	6.600	6.479	27.027	7.804	10.840	9.596	16.056	32.541	22.181
DEA	7.158	4.184	3.867	0.485	0.346	0.429	0.350	1.262	0.656	0.878	3.196	1.872	2.142	1.546	1.722	1.217	2.076	1.982	1.683	2.973	1.992	2.653
MEA	40.349	4.530	5.486	3.731	0.162	0.642	0.125	1.425	0.292	0.327	3.038	2.621	3.201	1.987	2.436	0.591	3.042	1.938	2.286	2.875	1.363	2.318
TEA	2.851	3.986	3.157	0.548	0.638	0.578	0.687	1.257	1.283	1.801	3.729	1.585	1.786	1.471	1.540	2.456	1.810	2.435	1.618	3.700	3.385	3.723

Table S3

anion / cation	NO3	HSO4	H2PO4	HCO3	Frc	Ac	SCN	Prp	Leu	BEN	OTF	DMP	2FPhO	4FPhO	3FPhO	2-CP	BF4	4-CP	3-CP	Tf2n	BCN4	PF6
N2222	1.7955	1.0637	0.9916	1.6531	2.1644	1.8246	1.7657	1.4462	1.4047	1.6127	1.4666	1.4916	1.6138	1.6149	1.6177	1.6638	1.3784	1.6435	1.6623	2.1397	2.4584	1.5442
P2221	1.6766	1.0172	0.9123	1.4895	1.9389	1.6722	1.7345	1.3758	1.3628	1.5684	1.4522	1.4615	1.6010	1.6004	1.6111	1.6639	1.3643	1.6404	1.6642	2.1802	2.5364	1.6180
N1111OH	0.8059	0.3252	0.9421	2.0724	2.7384	1.4983	0.3892	0.7780	0.6775	0.6679	0.3303	0.7492	0.5518	0.6016	0.5649	0.6250	0.1679	0.6786	0.6736	1.6973	1.2419	0.2550
P4444	1.9355	1.5289	0.9614	1.3082	1.5730	1.4870	2.4102	1.4774	1.5919	1.7906	1.8618	1.7694	1.8966	1.8277	1.8904	1.9619	2.1868	1.8679	1.9167	2.2271	2.9666	2.5260
MMIM	1.0093	0.4815	0.8338	1.5712	2.0673	1.4443	0.6762	0.9391	0.8584	0.8618	0.6707	0.9270	0.8632	0.9114	0.8894	0.9359	0.4551	0.9700	0.9756	2.0275	1.4821	0.6295
P4442	1.8358	1.3897	0.8921	1.2428	1.5170	1.4378	2.2824	1.4258	1.5372	1.7496	1.8328	1.7157	1.8852	1.8276	1.8917	1.9691	2.0800	1.8831	1.9354	2.3430	3.0638	2.5543
N4444	1.8290	1.4382	0.8892	1.2042	1.4391	1.3556	2.3228	1.3537	1.4752	1.6823	1.7868	1.6603	1.8213	1.7524	1.8224	1.9031	2.1273	1.8047	1.8600	2.2975	3.0444	2.5836
N11116	1.5904	1.3870	0.8675	1.0197	1.1140	1.0580	2.2254	1.1035	1.2115	1.4925	1.7954	1.3041	1.8759	1.8395	1.9311	2.0334	2.2685	1.9436	2.0187	3.0009	3.7065	3.4485
EMIM	0.8054	0.4549	0.5499	0.9035	1.1820	1.0196	0.7386	0.8410	0.8392	0.8958	0.8193	0.9406	0.9912	1.0275	1.0327	1.0954	0.6118	1.1074	1.1298	2.2674	1.9203	1.1325
P4442OH	1.2712	1.0199	0.6253	0.8319	1.0007	0.9801	1.6741	1.0057	1.1112	1.2819	1.3221	1.2561	1.4147	1.3804	1.4293	1.5039	1.7547	1.4385	1.4794	2.8596	3.1924	3.7358
P4442COOH	0.9535	0.8072	0.4956	0.5994	0.6843	0.6847	1.4004	0.7395	0.8441	1.0178	1.1038	0.9574	1.2839	1.2694	1.3279	1.4227	1.5290	1.3613	1.4113	3.6886	3.4876	5.8730
N1111COOH	0.1503	0.0772	0.2237	0.4064	0.5491	0.4995	0.1125	0.4685	0.4839	0.4613	0.2689	0.5805	0.4911	0.5519	0.5521	0.6539	0.0598	0.7259	0.7489	2.7436	1.7579	1.0823
HMIM	0.1358	0.0633	0.1726	0.3223	0.4617	0.4369	0.1057	0.4215	0.4581	0.4148	0.2969	0.5629	0.4934	0.5526	0.5607	0.6555	0.0725	0.7173	0.7477	2.4648	1.8689	1.0130
TEA	0.0373	0.0240	0.0604	0.0952	0.1335	0.1799	0.0483	0.2540	0.3091	0.2838	0.1591	0.4357	0.3359	0.3829	0.3954	0.4924	0.0268	0.5468	0.5742	1.9287	1.5053	0.8959
DEA	0.0085	0.0065	0.0135	0.0199	0.0315	0.0646	0.0262	0.1383	0.1939	0.1953	0.1386	0.3010	0.3368	0.4052	0.4514	0.5822	0.0228	0.6682	0.7376	2.5352	2.5705	6.4038
MEA	0.0010	0.0013	0.0020	0.0028	0.0053	0.0213	0.0605	0.0782	0.1243	0.1522	0.1568	0.2033	0.4712	0.6234	0.7781	1.0040	1.1454	1.2414	1.4648	4.0289	7.0164	232.2792

Figs

Fig. S1 ^{1}H NMR spectra of [MEA][Frc]

Fig. S2 ^{1}H NMR spectra of [MEA][Ac]

Fig. S3 ^{1}H NMR spectra of [MEA][Prp]

Fig. S4 ^{1}H NMR spectra of [DEA][Ac]

Fig. S5 ^{1}H NMR spectra of [TEA][Ac]

Fig. S6 ^{1}H NMR spectra of fresh and recycled [MEA][Ac]

Fig. S1

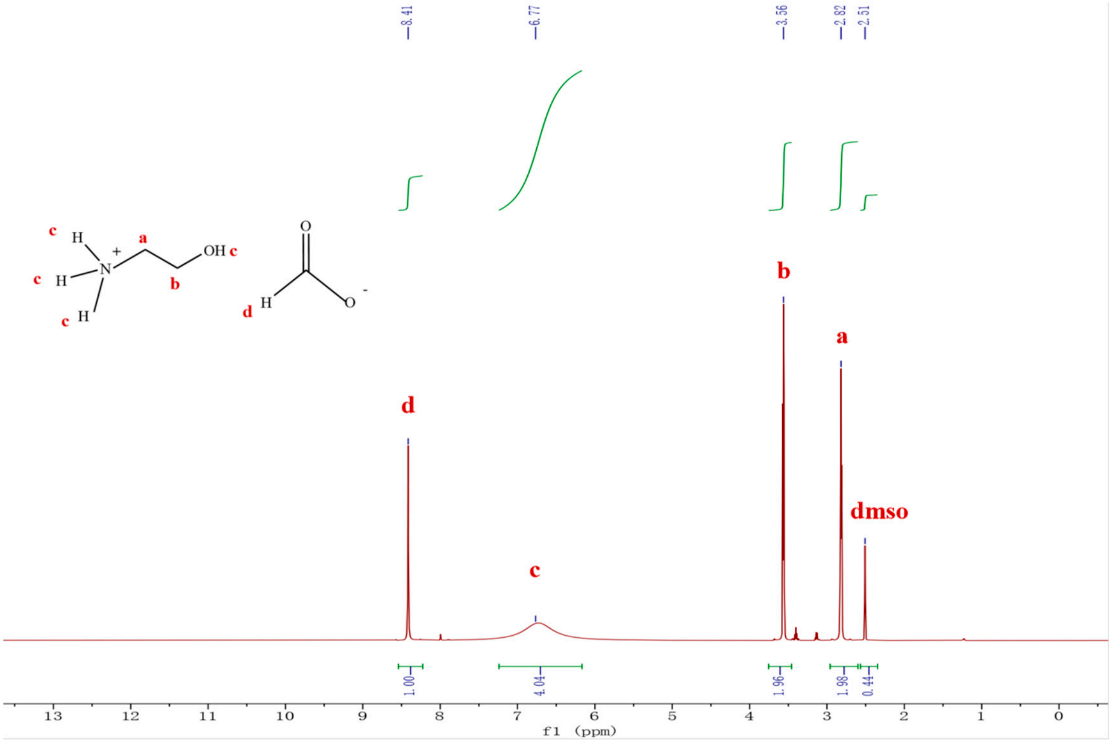

Fig. S2

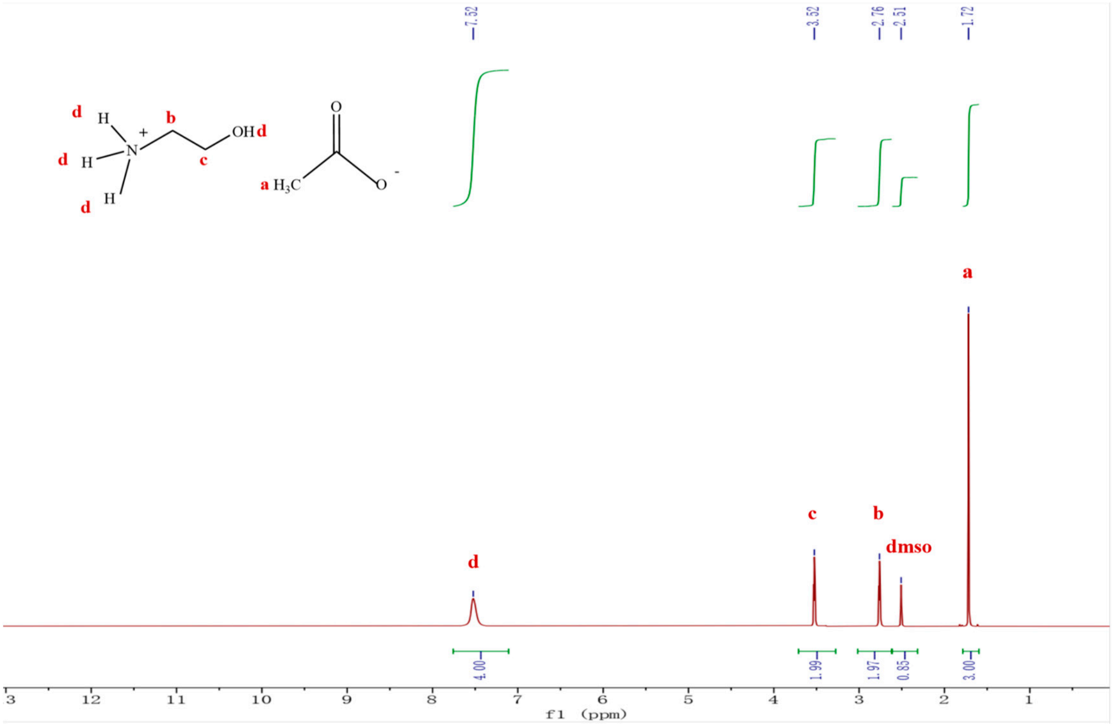

7.52
3.52
2.76
2.51
1.72
d
b
OH d
c
a
O
d
c
b
dmso
a
4.00
1.99
1.97
0.85
3.00
f1 (ppm)

Fig. S3

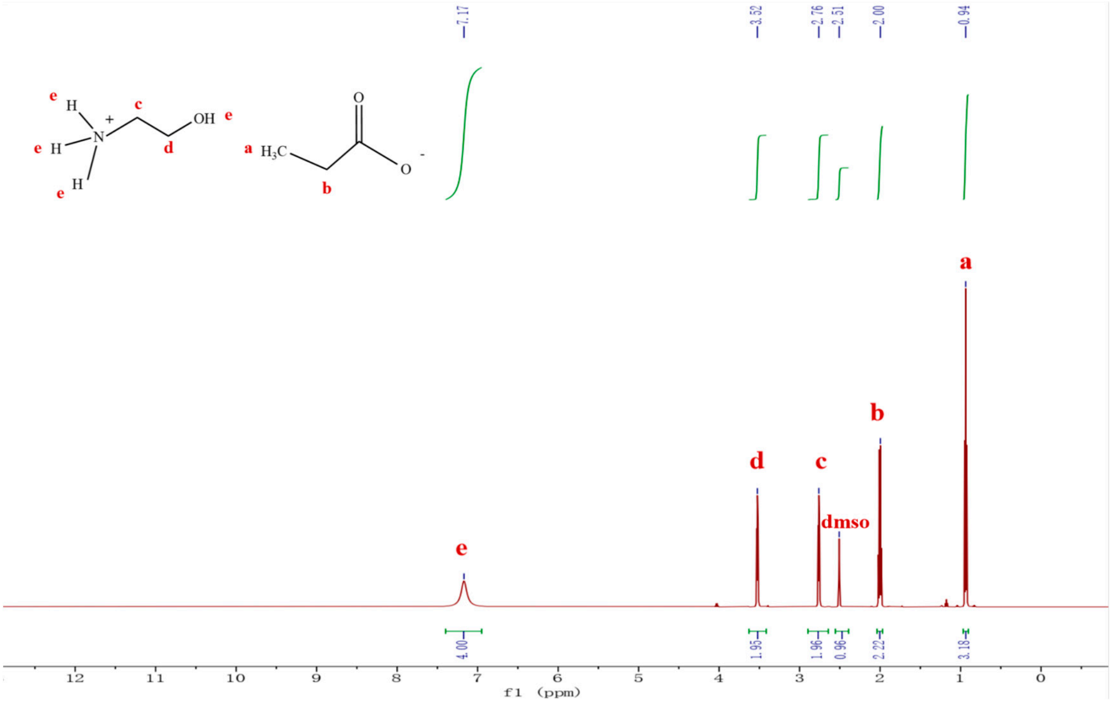

7.17
3.52
2.76
2.51
2.00
0.94
e
c
OH e
d
a
b
O
a
b
d
c
dmso
e
4.00
1.95
1.96
0.96
2.22
3.18
f1 (ppm)

Fig. S4

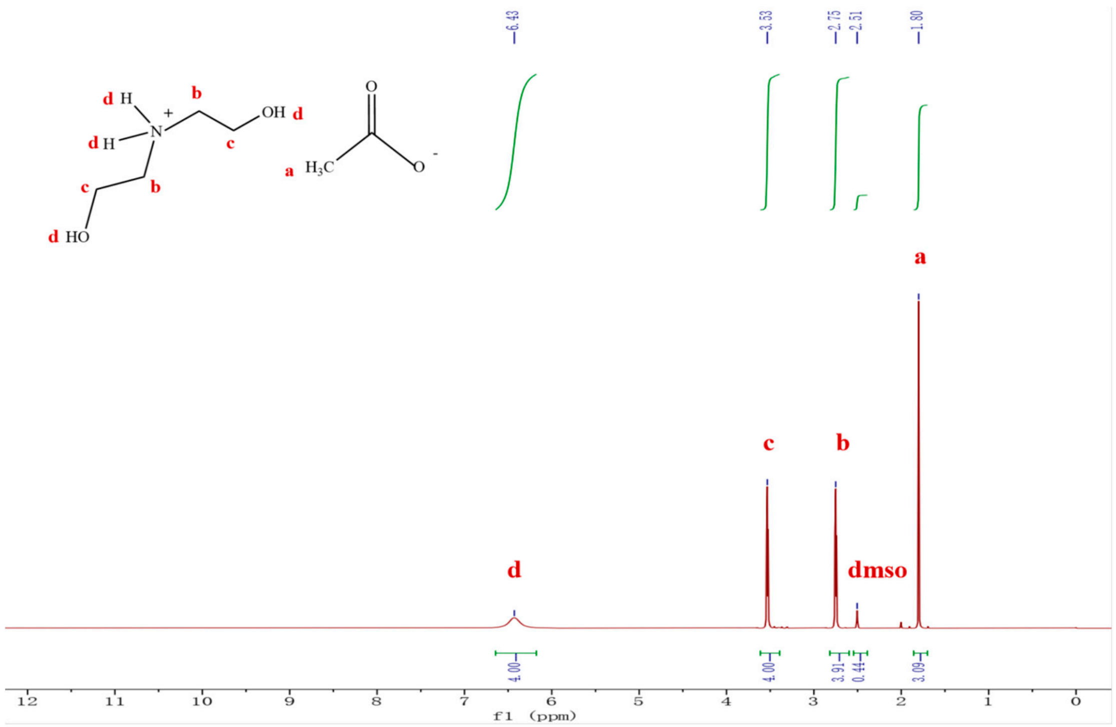

d H
b
OH d
d H
N
c
c
b
d HO
O
a H3C
O
6.43
3.53
2.75
2.51
1.80
a
c
b
d
dmso
4.00
4.00
3.91
0.44
3.09
12
11
10
9
8
7
6
5
4
3
2
1
0
f1 (ppm)

Fig. S5

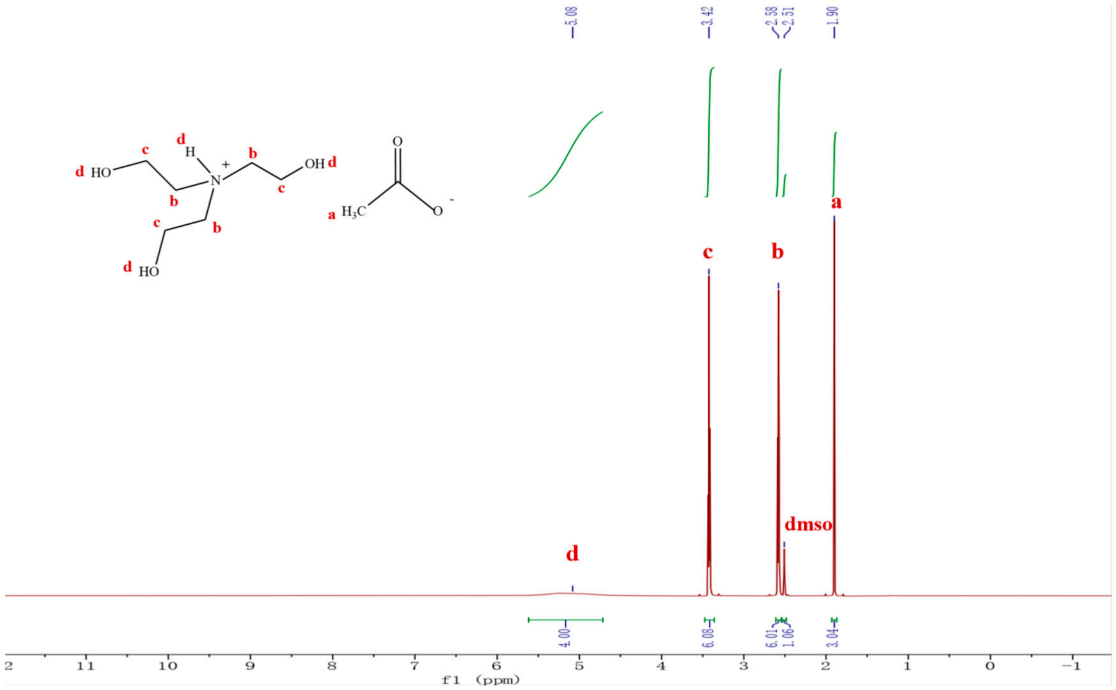

d HO
c
d
H
b
OH d
N
b
c
c
b
d HO
O
a H3C
O
5.08
3.42
2.58
2.51
1.90
a
c
b
dmso
d
4.00
6.08
6.01
1.06
3.04
2
11
10
9
8
7
6
5
4
3
2
1
0
−1
f1 (ppm)

Fig. S6

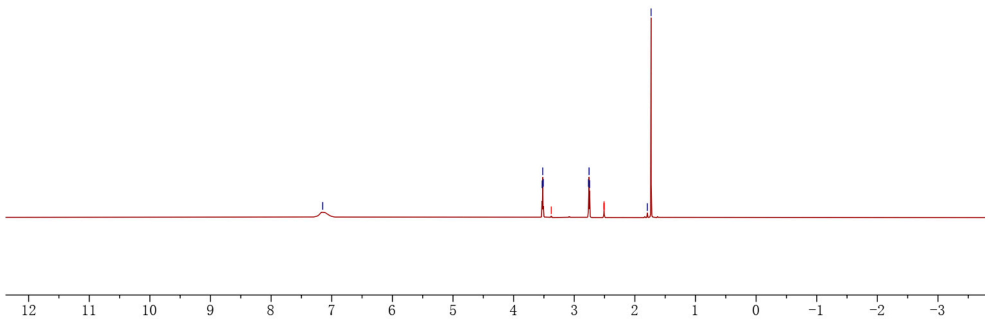
12 11 10 9 8 7 6 5 4 3 2 1 0 −1 −2 −3

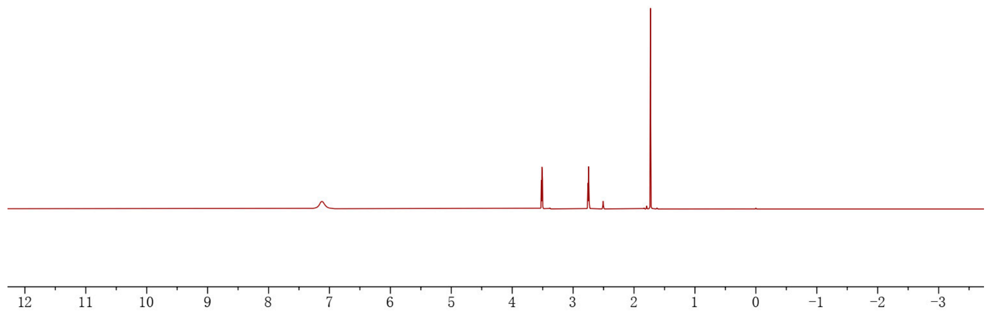
12 11 10 9 8 7 6 5 4 3 2 1 0 −1 −2 −3
